# Electronic Health Record Data for Lyme Disease Surveillance, Massachusetts, USA, 2017–2018

**DOI:** 10.3201/eid3007.230942

**Published:** 2024-07

**Authors:** Kshema Nagavedu, Karen Eberhardt, Sarah Willis, Monica Morrison, Aileen Ochoa, Susan Soliva, Sarah Scotland, Noelle M. Cocoros, Myfanwy Callahan, Liisa M. Randall, Catherine M. Brown, Michael Klompas

**Affiliations:** Harvard Medical School and Pilgrim Health Care Institute, Boston, Massachusetts, USA (K. Nagavedu, S. Willis, A. Ochoa, N.M. Cocoros, M. Klompas);; Massachusetts Department of Public Health, Boston (K. Nagavedu, M. Morrison, S. Soliva, S. Scotland, L.M. Randall, C.M. Brown);; Commonwealth Informatics, Waltham, Massachusetts, USA (K. Eberhardt);; Atrius Health, Boston (M. Callahan); Brigham and Women’s Hospital, Boston (M. Klompas)

**Keywords:** Lyme disease, zoonoses, public health surveillance, electronic health records, Massachusetts, United States, bacteria, vector-borne infections, ticks, Borrelia burgdorferi

## Abstract

Lyme disease surveillance based on provider and laboratory reports underestimates incidence. We developed an algorithm for automating surveillance using electronic health record data. We identified potential Lyme disease markers in electronic health record data (laboratory tests, diagnosis codes, prescriptions) from January 2017–December 2018 in 2 large practice groups in Massachusetts, USA. We calculated their sensitivities and positive predictive values (PPV), alone and in combination, relative to medical record review. Sensitivities ranged from 57% (95% CI 47%–69%) for immunoassays to 87% (95% CI 70%–100%) for diagnosis codes. PPVs ranged from 53% (95% CI 43%–61%) for diagnosis codes to 58% (95% CI 50%–66%) for immunoassays. The combination of a diagnosis code and antibiotics within 14 days or a positive Western blot had a sensitivity of 100% (95% CI 86%–100%) and PPV of 82% (95% CI 75%–89%). This algorithm could make Lyme disease surveillance more efficient and consistent.

Lyme disease, caused by infection with the bacterium *Borrelia burgdorferi*, is the most common vectorborne illness in the United States and is steadily affecting an expanding area of the country (*1*). In 2022, a total of 63,000 cases of Lyme disease were reported to US public health authorities (*2*). However, that case count was derived from provider-based and laboratory-based disease reports that likely underestimate the true burden of Lyme disease (*3*,*4*). Analysis of US insurance claims data suggests the true incidence of Lyme disease may be 6-fold to 8-fold higher than the number of cases reported to public health agencies (*4*).

Historically, the Massachusetts Department of Public Health (MDPH), as in most US states, required providers and laboratories to report information on suspected cases of Lyme disease. Laboratories sent positive test results, usually electronically, to MDPH, which then triggered MDPH to mail a request to providers for more information. Providers were asked to complete case report forms with the clinical information needed to classify each case in accordance with Council of State and Territorial Epidemiologists (CSTE) case definitions for Lyme disease (*5*). In 2016, given the high volume of positive Lyme disease–related laboratory tests in Massachusetts, the burden on public health staff to send case report forms to providers, the burden on clinicians to complete and return forms, and the burden on public health staff to abstract and compile case report forms, Massachusetts stopped mailing case report forms to providers after a positive laboratory result. In 2021, CSTE adopted an updated Lyme disease surveillance case definition that relies on laboratory criteria alone that does not require clinicians to complete supplementary case reports to classify suspected and probable cases in high-incidence states. This case definition went into effect nationally in January 2022 (*6*).

MDPH still receives electronic laboratory reports on Lyme disease, but they provide an incomplete picture of Lyme disease for several reasons: some cases of Lyme disease are treated empirically without testing; laboratory testing for early Lyme disease is insensitive; some Lyme disease laboratory tests are not specific; most laboratory tests do not differentiate between current active Lyme disease versus remote resolved Lyme disease; and electronic laboratory reporting does not include relevant contextual information, such as stage of disease and whether or what treatment was given. Those data could help provide an expanded picture of Lyme disease epidemiology to inform state and local policies and priorities as they pertain to Lyme disease prevention and management.

Using electronic health record (EHR) data provides a potential complementary strategy for Lyme disease surveillance. EHRs contain a wealth of clinical information on patients, including demographic data, vital signs, pregnancy status, clinical manifestations of disease, laboratory test orders, laboratory test results, and medication prescriptions. Automated analyses of these data can result in more complete and clinically detailed case reporting than provider- or laboratory-based reporting alone (*7*). However, information on how best to detect Lyme disease using EHR data is limited (*8*).

We sought to develop an algorithm for automated surveillance of Lyme disease using structured clinical data routinely recorded in EHRs. Potential components of a Lyme disease algorithm available in EHRs include diagnosis codes, laboratory tests, and prescriptions for medications typically used to treat Lyme disease (*8*). Those elements can be used as standalone criteria or in combination and likely vary in their sensitivity and positive predictive value (PPV). We assessed the frequency of those potential Lyme disease markers using EHR data from 2 large clinical practice groups and calculated the sensitivity and PPV of each marker, both alone and in combination, relative to medical record review. We then proposed a combination surveillance algorithm designed to maximize sensitivity and PPV and validated its performance in a third, independent practice group. The Institutional Review Board of the Harvard Pilgrim Health Care Institute reviewed this study and deemed it public health operations.

## Methods

### Data Sources

We selected potential markers of Lyme disease in EHR data through consultation with MDPH epidemiologists and an infectious disease physician. Those markers included diagnosis codes from the International Classification of Diseases, 10th Revision, Clinical Modification (ICD-10-CM), as well as positive laboratory tests for Lyme disease and prescriptions for antibiotics typically used to treat Lyme disease, excluding postexposure prophylaxis after tick bites (Appendix, Tables 1–3).

We identified patients who had >1 potential Lyme disease marker during the study period of January 1, 2017–December 31, 2018, in 3 large clinical practice groups located in eastern Massachusetts using the Electronic Medical Record Support for Public Health Surveillance platform (ESP; https://www.esphealth.org) (*9*–*13*). ESP is an open-source public health surveillance platform that uses daily extracts of data from EHR systems to identify and report conditions of public health interest to health departments. ESP maps EHR data to common terms, analyzes the data for reportable diseases, and automatically submits case reports to health departments’ electronic surveillance systems or generates aggregate summaries. At the time of our study, ESP captured ≈50% of the population of Massachusetts for reportable infectious disease cases and ≈20% of the population for chronic disease surveillance.

The 3 practice groups included in the evaluation were Boston Medical Center, Cambridge Health Alliance, and Atrius Health. Boston Medical Center is a 514-bed academic medical center in the city of Boston that provides inpatient, emergency, and outpatient care to ≈220,000 persons. Cambridge Health Alliance is a safety net system for vulnerable populations living in communities north of Boston and provides inpatient, emergency, and outpatient care for ≈200,000 persons. Atrius Health provides outpatient care to a generally well-insured population of ≈700,000 persons primarily in eastern Massachusetts. We used the data from Boston Medical Center and Cambridge Health Alliance to develop a surveillance algorithm and data from Atrius Health to validate results.

### Algorithm Development

We calculated the sensitivities and positive predictive values of each EHR-based potential Lyme disease marker and combinations of these markers for 2017–2018 among patients seen at Boston Medical Center and Cambridge Health Alliance. We created 10 nonoverlapping strata with unique combinations of potential algorithm components (diagnosis codes, medications, positive enzyme immunoassays, and positive Western blots for both IgG and IgM) (Table 1). We then reviewed 209 randomly selected charts and arrayed them into the strata.

**Table 1:** Sensitivity and positive predictive values of candidate criteria for a Lyme disease algorithm at 2 clinical practice groups in Massachusetts, USA, 2017–2018*

Strata	Algorithm components					Charts reviewed/ flagged			No. Lyme disease cases†				Summary performance		
	ICD codes	+ EIA	+ WB	ABX									PPV, % (95% CI)‡	No. projected Lyme disease cases§	Sensitivity, % (95% CI)#
						Site 1	Site 2		C	P	S				
1	1					18/90	20/52		3	1	0		10.5 (1.5–21.6)	14.9	6.5 (1.7–13.2)
2	1			1		20/53	20/105		22	3	0		62.5 (47.4−77.0)	98.8	43.1 (35.8–49.2)
3		1				12/12	19/65		0	0	1		3.2 (0.0–12.0)	2.5	1.1 (0.0–4.3)
4	1	1				0	1/3		0	0	0		0.0 (0.0–85.0)	0.0	0.0 (0.0 −1.3)
5		1		1		5/5	12/18		0	0	0		0.0 (0.0–10.4)	0.0	0.0 (0.0–1.2)
6	1	1		1		2/2	7/11		2	0	0		22.2 (0.0–51.8)	2.9	1.3 (0.0 −3.0)
7		1	1			9/9	15/15		2	6	16		100.0 (92.5–100.0)	24.0	10.5 (8.3–10.9)
8	1	1	1			6/6	3/5		1	3	5		100.0 (81.5–100.0)	11.0	4.8 (3.7–5.4)
9		1	1	1		12/15	5/9		1	9	7		100.0 (89.6–100.0)	24.0	10.5 (8.8–11.8)
10	1	1	1	1		8/29	15/22		14	5	4		100.0 (92.2–100.0)	51.0	22.3 (19.2–25.1)

*ABX, antibiotics; C, confirmed; EIA, enzyme immunoassay; ICD, International Classification of Diseases, 10th Revision, Clinical Modification; P, probable; PPV, positive predictive value; S, suspected; WB, Western blot. †Based on criteria from the Council of State and Territorial Epidemiologists and Centers for Disease Control and Prevention. ‡PPV for each stratum was calculated by summing up the total number of confirmed, probable, and suspected cases for the stratum and then dividing by the total number of charts reviewed for the stratum. §The total number of projected Lyme disease cases for each stratum was calculated by multiplying the total number of charts flagged in the stratum by the positive predictive value for the stratum. #Sensitivity was calculated as the total number of projected Lyme disease cases for the criteria of interest divided by the total number of projected Lyme disease cases for the entire population. The total number of projected Lyme disease cases for the entire population is 229.1, which is the sum of the projected Lyme disease cases from each of the 10 unique strata in the top half of the table using unrounded numbers.

We conducted chart reviews in 2020 and 2021 using standardized forms to capture information in the EHR on erythema migrans; tick bite or exposure to ticks; signs and symptoms associated with Lyme disease; cardiovascular, musculoskeletal, or nervous system manifestations of Lyme disease; prescriptions for antibiotics used to treat Lyme disease; and results of Lyme disease–related laboratory tests (enzyme immunoassays and Western blots). Each case was adjudicated using the 2017 CSTE surveillance definition for Lyme disease and classified as confirmed, probable, or suspected Lyme disease, prophylaxis for Lyme disease, or not a case (*5*). MDPH personnel performed record review, data abstraction, and adjudication; they received training on the abstraction forms before their first medical record review. Records were single adjudicated.

We calculated the PPV for each stratum relative to 2017 CSTE criteria as the number of confirmed, probable, or suspected cases in the chart review sample for the stratum divided by the number of charts reviewed in the stratum. We multiplied the count of patients in each stratum by the stratum PPV to project the total number of patients with Lyme disease in that stratum. We then summed the projected number of patients with Lyme disease from each stratum to estimate the total number of Lyme disease patients in the study population. We used that estimate of the total number of Lyme disease patients as the denominator for calculating the sensitivity of each stratum and the sensitivities of all candidate Lyme disease surveillance criteria.

We estimated PPVs and sensitivities for all candidate criteria (e.g., ICD code, enzyme immunoassay, ICD code and antibiotics, etc.) by combining the counts of charts flagged, charts reviewed, and the number of patients with confirmed, probable, or suspected Lyme disease from each of the strata that included the candidate criteria of interest (Table 2). We calculated PPV as the number of persons with confirmed, probable, or suspected Lyme disease divided by the number of charts reviewed for each candidate criteria. We calculated sensitivity by multiplying the number of persons flagged by the candidate criteria PPV to project the total number of persons in the study population with the candidate criteria and then dividing by the estimated total number of Lyme disease patients in the total study population as described above.

**Table 2:** Summary performance rates for algorithm components and combinations calculated by summing the pertinent strata in study of electronic medical records for Lyme disease, Massachusetts, 2017–2018*

Strata used	Algorithm components	Charts reviewed/ flagged			No. Lyme disease cases†				Summary performance		
									PPV, % (95% CI)‡	No. projected Lyme disease cases§	Sensitivity, % (95% CI)#
		Site 1	Site 2		C	P	S				
1, 2, 4, 6, 8, 10	ICD code	54/180	66/198		42	12	9		52.5 (43.6–61.4)	198.5	86.6 (69.8–100)
3–10	Positive EIA	54/78	77/148		20	23	33		58.0 (49.5–66.4)	131.1	57.2 (46.7–68.8)
2, 6, 10	ICD code and antibiotics	30/84	42/138		38	8	4		69.4 (58.6–79.8)	154.2	67.3 (54.8–81.7)
5, 6, 9, 10	Positive EIA and antibiotics	27/51	39/60		17	14	11		63.6 (51.9–75.0)	70.6	30.8 (24.4–38.2)
5, 6, 7–10	(Positive EIA and antibiotics) or positive WB	42/66	57/80		20	23	32		75.8 (67.1–83.9)	110.6	48.3 (40.2–56.5)
2, 6, 7–10	(ICD and antibiotics) or positive WB	57/114	65/167		42	26	32		82.0 (74.9–88.5)	230.3	100 (86.2–100)

*Strata and algorithms are defined in Table 1. ABX, antibiotics; C, confirmed; EIA, enzyme immunoassay; ICD, International Classification of Diseases, 10th Revision, Clinical Modification; P, probable; PPV, positive predictive value; S, suspected; WB, Western blot. †Based on criteria from the Council of State and Territorial Epidemiologists and Centers for Disease Control and Prevention. ‡PPV value for each stratum was calculated by summing up the total number of confirmed, probable, and suspected cases for the stratum and then dividing by the total number of charts reviewed for the stratum. §The total number of projected Lyme disease cases for each stratum was calculated by multiplying the total number of charts flagged in the stratum by the positive predictive value for the stratum. #Sensitivity was calculated as the total number of projected Lyme disease cases for the criteria of interest divided by the total number of projected Lyme disease cases for the entire population. The total number of projected Lyme disease cases for the entire population is 229.1, which is the sum of the projected Lyme disease cases from each of the 10 unique strata in the top half of the table using unrounded numbers.

We validated the final algorithm by applying it to 2017–2018 EHR data drawn from Atrius Health. To maximize the efficiency of chart reviews, we opted to review the charts of 25 randomly selected patients that met the final algorithm’s diagnosis code and antibiotic criteria who did not have positive Western blots and then assumed all other patients flagged by the final algorithm who did have positive Western blots were true positive cases. We conducted data analyses using SAS version 9.4 (SAS Institute Inc., https://www.sas.com).

## Results

### Algorithm Validation

No single criterion had optimal sensitivity and positive predictive value (Table 1). Sensitivity ranged from 57.2% (95% CI 46.7%–68.8%) for Lyme disease enzyme immunoassay (EIA) to 86.6% (95% CI 69.8%–100%) for a Lyme disease diagnosis code. Conversely, PPVs ranged from 58.0% (95% CI 49.5%–66.4%) for Lyme disease EIA to 52.5% (95% CI 43.6%–61.4%) for a Lyme disease diagnosis code. Combining criteria, however, improved performance. The combination of a Lyme disease diagnosis code and a prescription for an antibiotic of interest within 14 days had a sensitivity of 67.3% (95% CI 54.8%–81.7%) and PPV of 69.4% (95% CI 58.6%–79.8%). The combination of Lyme disease EIA and antibiotics had a sensitivity of 30.8% (95% CI 24.4%–38.2%) and a PPV of 63.6% (95% CI 51.9%–75.0%). A multicomponent algorithm composed of a Lyme disease diagnosis code and a prescription within 14 days or a positive Western blot had a sensitivity of 100% (95% CI 86.2%–100%) and PPV of 82.0% (95% CI 74.9%–88.5%).

We validated the multicomponent algorithm in Atrius Health. Chart reviews at Atrius focused on the combination of a Lyme disease diagnosis code and an antibiotic within 14 days. We otherwise assumed all patients with positive Western blots to have confirmed Lyme disease. On this basis, we estimated the PPV of the combination of a Lyme disease diagnosis code and antibiotics within 14 days, or a positive Lyme disease Western blot as 90.0% (95% CI 87.0%–93.0%).

We applied the algorithm to retrospective data within from 3 clinical practices that collectively provide care for >20% of the state population. For June–August 2022, we found that the prevalence of Lyme disease was 1 case/1,000 patients (*14*). Patients were 71% Caucasian and 53% male. Cases were clustered in neighborhoods to the south and north of Boston as well as on Cape Cod and the surrounding islands. Our results were consistent with historic data on the geographic distribution of Lyme disease in Massachusetts (*15*).

## Discussion

In this analysis of EHR-based algorithm criteria for Lyme disease, we observed that a diagnosis code for Lyme disease and a prescription for a relevant antibiotic within 14 days, or a positive Western blot was associated with high sensitivity (100%) and PPV (82%) for chart review–confirmed Lyme disease in accordance with CSTE criteria.

A key challenge with Lyme disease surveillance using EHR data is that no 1 criterion is both sensitive and specific. Diagnosis codes are variably assigned to patients and do not reliably differentiate between current acute disease versus remote resolved disease. Combining this criterion with an antibiotic prescription, however, increased positive predictive value. Likewise, surveillance using Lyme disease test results alone is imperfect. A first-tier Lyme disease EIA is prone to false positives and misses infections diagnosed clinically and treated empirically without testing. Indeed, Lyme disease guidelines recommend treating patients in disease-endemic areas who have a classic erythema migrans rash without performing any laboratory tests (*16*). Likewise, focusing surveillance on second-tier Western blots alone is specific but misses patients for whom the Western blot is not ordered.

At the time this work was done, participating practices exclusively used Western blots as second-tier tests after a positive or equivocal first-tier EIA. In July 2019, the US Food and Drug Administration approved Lyme disease assays that use an EIA rather than a Western blot as a second-tier test (*17*). Although our chart review did not assess this modified 2-tiered testing algorithm, 2 positive EIA results from a single collection date of an FDA-cleared assay is likely an acceptable alternative to a positive Western blot in a Lyme disease surveillance algorithm.

Strengths of our analysis include the use of detailed EHR data (such as diagnosis codes, test results, and antibiotic prescriptions) to enhance Lyme disease surveillance beyond what is possible using diagnosis codes or laboratory test results alone; the capacity to identify early cases of Lyme disease among persons who were untested or who had negative tests, as long as their clinicians assigned a diagnosis code for Lyme disease and prescribed antibiotics; the derivation of an algorithm using data from 2 independent practice groups and validation in an independent third group; and the use of structured chart reviews to apply CSTE Lyme disease criteria. Limitations of our analysis include limited sampling per criterion, which led to wide CIs per criterion; our dependence on retrospective chart reviews to apply CSTE criteria and thus the possibility of misclassification resulting from incomplete or inaccurate documentation; and our focus on 3 practice groups in 1 high-incidence state, which may limit the generalizability of our findings, particularly to areas with less endemic disease. Likewise, our medication criterion did not incorporate dose or duration, which may have decreased specificity.

In Massachusetts, Lyme disease is endemic; traditional surveillance methods have been burdensome and incomplete. The EHR-based algorithm for Lyme disease surveillance complemented traditional surveillance methods for tracking disease incidence. Updating and revalidating the surveillance algorithm to include the FDA-cleared modified 2-tier laboratory test type will further strengthen the algorithm (*14*). Adopting the algorithm for routine reporting through ESP will provide DPH with real-time data on the incidence, temporal change, geographic distribution, and demographic characteristics of Lyme disease in the state.

Our analysis demonstrates the potential value of EHR-based algorithms for public health surveillance relative to electronic laboratory reporting alone because of the capacity to integrate diagnosis codes and prescriptions along with diagnostic testing. The method can readily be extended to provide surveillance for other tickborne infections and co-infections, such as babesiosis and anaplasmosis. This method might also be usable for surveillance of other complex conditions without definitive diagnostic tests or biomarkers, such as myalgic encephalomyelitis or postacute sequelae of COVID-19.

Appendix Additional information about the use of electronic medical data for Lyme disease surveillance, Massachusetts, USA, 2017–2018.
