# Supplementary material for: Electronic Health Record Data for Lyme Disease Surveillance, Massachusetts, USA, 2017–2018
Source: Emerg Infect Dis. 2024 Jul;30(7):1374–9. doi: 10.3201/eid3007.230942 (PMC11210632; doi:10.3201/eid3007.230942)
Supplement: Appendix — Additional information about the use of electronic medical data for Lyme disease surveillance, Massachusetts, USA, 2017–2018. [file 23-0942-Techapp-s1.pdf]

Article DOI: http://doi.org/10.3201/eid3007.230942

EID cannot ensure accessibility for supplementary materials supplied by authors. Readers who have difficulty accessing supplementary content should contact the authors for assistance.

Electronic Health Record Data for Lyme Disease Surveillance, Massachusetts, 2017–2018

Appendix

Appendix Table 1. Diagnosis codes used to identify Lyme disease*

Code type	Code	Description
ICD-10-CM	A69.20	Lyme disease, unspecified
ICD-10-CM	A69.21	Meningitis due to Lyme disease
ICD-10-CM	A69.22	Other neurologic disorders in Lyme disease
ICD-10-CM	A69.23	Arthritis due to Lyme disease
ICD-10-CM	A69.29	Other conditions associated with Lyme disease

ICD-10-CM, International Classification of Diseases, 10th revision, clinical modification

Appendix Table 2. Lyme medications

Medication name
Doxycycline
Amoxicillin
Cefuroxime
Ceftriaxone
Cefotaxime
Azithromycin
Tetracycline

Appendix Table 3. Laboratory tests

Component Name	LOINC	LOINC Name
IGM EIA	16482–2	Borrelia burgdorferi Ab.IgM: ACnc: Pt: Flu: Qn: EIA
IGG EIA	16481–4	Borrelia burgdorferi Ab.IgG: ACnc: Pt: Flu: Qn: EIA
Lyme Western blot IgM	23982–2	Borrelia burgdorferi Ab.IgM: ACnc: Pt: Flu: Ord: IB
Lyme Western blot IgG	29898–4	Borrelia burgdorferi Ab.IgG: ACnc: Pt: xxx: Ord: IB
